# Case-based surveillance of measles in Sicily during 2012-2017: The changing molecular epidemiology and implications for vaccine strategies

**DOI:** 10.1371/journal.pone.0195256

**Published:** 2018-04-04

**Authors:** Fabio Tramuto, Carmelo Massimo Maida, Fanny Pojero, Giuseppina Maria Elena Colomba, Alessandra Casuccio, Vincenzo Restivo, Francesco Vitale

**Affiliations:** 1 Department of Health Promotion Sciences and Mother-Child Care “G. D’Alessandro” – Hygiene section, University of Palermo, Palermo, Italy; 2 Clinical Epidemiology Unit, University Hospital “Paolo Giaccone”, Palermo, Italy; Sun Yat-Sen University, CHINA

## Abstract

Following the indication of the World Health Organization, a national plan for the elimination of measles was approved in Italy and this included the improvement of the molecular surveillance of measles viruses and the interruption of indigenous transmission of the disease. Nevertheless, large outbreaks continue to occur in almost all regions of the country, including Sicily. Here we describe the epidemiology and molecular dynamics of measles viruses as a result of the measles surveillance activity carried out by the “Reference Laboratory for Measles and Rubella” in Sicily over a 5-year period. Biological samples of 259 suspected measles cases were tested for viral RNA detection and a total of 223 (86.1%) were classified as laboratory confirmed. The median age of confirmed measles cases was 21.0 years and about half of them were adults aged 19 years and older. Overall, one-third of the patients showed clinical complications and these latter were more common among adults than children (44.9% *vs*. 25.7%). The vast majority of measles cases were unvaccinated (94.2%, n = 210). The phylogenetic analysis of 221 measles virus nucleotide sequences revealed sporadic detections of genotypes D4 and H1, while endemic circulation of genotypes D8 and B3 was documented. Genotype D8 was associated with epidemics occurred between 2013 and 2016, whereas genotype B3 was more recently introduced into Sicily characterizing the current measles outbreak. The results of this study confirm the autochthonous co-circulation of viral variants belonging to different genotypes during the study period, and emphasizes the need of measles surveillance programmes in order to investigate the viral dynamics, the pathways of disease transmission, and to eventually adapt the development of successfull vaccine formulations.

## Introduction

Measles is a highly contagious human disease characterized by acute symptoms including high fever, conjunctivitis, coryza, cough and a maculopapular rash. It can lead to serious illness with severe complications, including pneumonia, encephalitis and death.

The World Health Organization (WHO) have launched a global strategic plan with the goal of improving the implementation of vaccination programmes for measles and rubella elimination [1]. This determined the interruption of transmission in the WHO region of the Americas [2], whereas the Eastern Mediterranean, European and Western Pacific WHO regions have formally set target dates for measles elimination [3].

Nevertheless, despite efforts and recommendations of the public health community, sustained outbreaks continue to occur also in countries which introduced mass vaccination schedules against measles, as part of their national immunization programmes, and an estimated 134,200 global deaths for measles were reported in 2015, mostly in low-income countries [4].

In Italy, measles infection must be mandatory notified to the Local Health Authority (LHA) within 12 hours from the onset of symptoms. LHA is responsible for the investigation and public health management of suspected cases and contacts, following the case definition of the European Union (EU) Commission Decision of 2012 [5]. A “National Plan for the Elimination of Measles and Congenital Rubella (PNEMoRc 2010–2015)” [6] was approved by the Italian Ministry of Health, substantially improving the molecular surveillance of measles and rubella. In accordance to the PNEMoRc [6], individual data and clinical details are collected together with biological specimens for laboratory confirmation of measles/rubella virus infection and genotyping. Molecular data are used to recognize authocthonous or imported cases and to identify epidemiologically-linked outbreaks.

In this context, Italy is one of the fourteen WHO European Region Member States where measles remains endemic [7] and, to date, about 5,000 cases have been reported since 1 January 2017 from almost all regions of the country [8], showing temporal patterns of genetic variation as documented with phylogenetic analyses of wild-type MVs circulating in Italy [9–11].

Measles vaccination has been implemented in Sicily, either in association with mumps and rubella (MMR) or combined with varicella (MMRV), following a two-dose schedule and it is strongly recommended for all infants between 13 and 15 months and for children at 5–6 years of age. Based on the available data from regional health authority, the proportion of children being vaccinated for MMR/MMRV has progressively fallen in recent years (coverage rate in 2016: 82.3%, range 69.5%—90.8%) and this has favoured the endemic circulation of measles virus (MV) in the general population.

Since 2012, a Sub-National Reference Laboratory affiliated to the Italian network MoRoNet [http://www.moronetlab.it] has been implemented in Sicily, in order to confirm the suspected cases, to molecularly characterize the genotype of identified viral strains, and to provide an insight into the local epidemiology.

This study aimed to report the epidemiology of measles infection in Sicily, describing the genetic diversity of circulating MVs and variants during the period 2012–2017 by using a phylogenetic approach.

## Materials and methods

### Study population, case definition and clinical samples

A retrospective study was carried out between March 2012 and August 2017 in Sicily, an Italian administrative region which accounts for more than 5 million inhabitants and includes nine provinces (Agrigento, Caltanissetta, Catania, Enna, Messina, Palermo, Ragusa, Siracusa, and Trapani).

According to PNEMoRc, the LHA epidemiology unit of each province contributed to collect oral fluid and/or urine specimens from each suspected measles case within 7 days after the onset of rash [12]. Biological samples were transported to the reference laboratory, under appropriate conditions, and analysed with all available clinical data.

All suspected cases were notified by general practitioners (community cases) or by hospital infectious disease units throughout the region, and a confirmed measles case was defined as any person meeting both the clinical and laboratory criteria. That means an association of fever, a generalized maculopapular rash, one symptom among cough, coryza, and conjunctivitis, together with laboratory positive finding for measles IgM serology and/or viral RNA in oral fluid/urine samples. Individuals who did not fulfill the case definition were reported as “not confirmed”.

The present work was reviewed and approved by the institutional review board of the University Hospital “A.O.U.P.–P. Giaccone” of Palermo (Sicily), health data were fully anonymized before accession and stored according to the Italian laws on privacy, and the research was conducted following the Helsinki declaration statements.

### Routine testing, measles virus genotyping and phylogenetic analysis of measles sequences

Viral RNA was extracted using QIAamp Viral RNA extraction kit (QIAGEN) according to the manufacturer’s suggested protocol, and viral RNA was eluted from the spin column in 60 μL of elution buffer. Eluted RNA was divided into aliquots and stored immediately at -80°C until further use. Each sample was tested by one-step real-time RT-PCR for the presence of MV-RNA [13], using a QuantStudio 7 Flex Real-Time PCR system (Applied Biosystems).

All real-time PCR-positive samples were amplified by end-point RT-PCR and genotyped using primers amplifying the N-terminal 450-nt fragment of the measles N gene, according to the WHO-approved sequencing methods [12,14,15].

RT-PCR products were purified, labeled with the Big Dye Terminator chemistry v3.1 (Applied Biosystems), and then analysed by an automatic genetic analyser ABI PRISM 3130xl (Applied Biosystems). Sequences were assembled with the software package CLC Main Workbench v7.6.13 and aligned with Clustal X [16]. Moreover, alignments were manually edited when needed using BioEdit v7.2.6 [http://www.mbio.ncsu.edu/BioEdit/bioedit.html].

The evolutionary model that best fitted the data was evaluated with JmodelTest v. 2.1.7 [17] and the general time reversible + gamma distribution (GTR + G) model of nucleotide substitution was selected.

Phylogenetic trees were drawn with MEGA 6 [18] by neighbor-joining using 1000 bootstrap replicates, including the WHO-designated reference sequences for each genotype and a representative set of sequences from different world countries.

All N gene sequences of MVs included in this study were named according to the official WHO nomenclature [19] and submitted to GenBank together with relevant epidemiological data.

Being the laboratory part of the national network MoRoNET, sequences were also shared with the National Laboratory for Measles and Rubella at the Italian National Institute of Health (Rome) that transmits all Italian sequences to the WHO Measles Nucleotide Surveillance (MeaNS) database.

### Data managements and statistical analysis

Descriptive statistics were used to summarize each of the socio-demographic and clinical variables included in the dataset (counts, percentages, median and interquartile range, as appropriate).

In accordance to the recommended MMR/MMRV immunization schedule in Sicily, the study population was subdivided into three different age-groups (≤1, 1–4, 5–9 years). Additionally, the age-groups 10–18, 19–29, and ≥30 years were used for further stratification of paediatric and adult populations.

Median values were compared using the Mann-Whitney U test, while the groups were compared using the chi-2 or Fisher’s exact test, as appropriate. Finally, univariate ORs and 95% confidence intervals (CIs) were calculated to measure the association between clinical complication and age-group using logistic regression model. All of the analyses with *p-*values of 0.05 or less were considered to be statistically significant (two tailed). Data were processed with the STATA MP statistical software package v14.2 for Apple (StataCorp).

## Results

### Epidemiology of measles in Sicily between 2012 and 2017

A total of 259 suspected cases were reported and analysed for confirmation of measles infection and genetic characterization of circulating MVs (Table 1); 86.1% (n = 223/259) were laboratory confirmed.

**Table 1:** Reported measles cases by health settings, Sicily, March 2012—August 2017.

	Total	Confirmed	*Health settings*	
			*Hospital*	*Community*
**Number of reported measles cases, n (%)**	**259**	**223 (86.1)**	161 (72.2)	62 (27.8)
**Sex**				
***Male***	129 (49.8)	111 (49.8)	79 (49.1)	32 (51.6)
***Female***	130 (50.2)	112 (50.2)	82 (50.9)	30 (48.4)
**Age [years; median (IQR)]**	14.0 (25.0)	21.0 (26.0)	13.0 (26.0) ^a^	24.5 (26.0) ^a^
*Age-group (years)*				
<1	13 (5.0)	11 (4.9)	6 (3.7)	5 (8.1)
1–4	71 (27.4)	55 (24.7)	50 (31.1)	5 (8.1)
5–9	28 (10.8)	21 (9.4)	14 (8.7)	7 (11.3)
10–18	25 (9.74)	18 (8.1)	14 (8.7)	4 (6.4)
19–29	64 (24.7)	62 (27.8)	40 (24.8)	22 (35.5)
≥30	58 (22.4)	56 (25.1)	37 (23.0)	19 (30.6)

^*a*^ *p* = 0.044

In general, a higher proportion of confirmed cases was found in hospitalized patients (72.2%; n = 161/223) when compared to the community setting (27.8%; n = 62/223). No gender differences were found in terms of reported or confirmed cases.

The median age of measles cases was 21.0 years (IQR = 26.0 years), subjects who required hospitalization were significantly younger than those reported in the community (median age: 13.0 years *vs*. 24.5 years; *p* = 0.044), whereas about half of hospitalized cases (47.8%; n = 77/161) were among adults aged 19 years and older (Table 1). All hospitalized patients recovered. In summary, S1 Fig depicts the geographic distribution of measles confirmed infections documented across the whole region.

As described in Table 2, most people who get the measles had mild illness (64.1%; n = 143/223), whereas, notably, adults were at higher risk than children of developing clinical complications (OR = 2.35; *p* = 0.003). Generally, diarrhoea and keratoconjuntivitis prevailed (40.0%; n = 32/80 and 32.5%; n = 26/80, respectively), one-quarter of complicated patients presented pneumonia (25.0%; n = 20/80), while about 20.0% developed laryngotracheobronchitis (18.8%; n = 15/80) or showed signs of hepatopathy (17.5%; n = 14/80). Stomatitis, thrombocytopenia, respiratory failure and otitis were found at lower frequencies.

**Table 2:** Laboratory confirmed measles cases by clinical complications and age-group, Sicily, March 2012—August 2017.

	Total	Age-group (years)	
		Children	Adults
**Number of confirmed measles cases, n (%)**	**223**	**105 (47.1)**	**118 (52.9)**
**Cases without complications (mild cases)**	143 (64.1)	78 (74.3)	65 (55.1)
**Cases with at least one complication** ^a^	80 (35.9)	27 (25.7) ^b^	53 (44.9) ^b^
*Diarrhoea*	*32 (40*.*0)*	*10 (37*.*0)*	*22 (41*.*5)*
*Keratoconjuntivitis*	*26 (32*.*5)*	*7 (25*.*9)*	*19 (35*.*8)*
*Pneumonia*	*20 (25*.*0)*	*6 (22*.*2)*	*14 (26*.*4)*
*Laryngotracheobronchitis*	*15 (18*.*8)*	*5 (18*.*5)*	*10 (18*.*9)*
*Hepatopathy*	*14 (17*.*5)*	*1 (3*.*7)*	*13 (24*.*5)*
*Stomatitis*	*10 (12*.*5)*	*2 (7*.*4)*	*8 (15*.*1)*
*Thrombocytopenia*	*6 (7*.*5)*	*0*	*6 (11*.*3)*
*Respiratory failure*	*6 (7*.*5)*	*2 (7*.*4)*	*4 (7*.*5)*
*Otitis*	*5 (6*.*2)*	*0*	*5 (9*.*4)*
*Other* ^c^	*6 (7*.*5)*	*4 (14*.*8)*	*2 (3*.*8)*

Children, ≤18 years old; adults: >18 years old.

^*a*^ Complications are listed according to the PNEMoRc 2010–2015 [6]. Individual cases could have multiple complications and the percentages of each complication are calculated using as the denominator the total number of measles cases with at least one complication, n = 80.

^*b*^ Odds ratio = 2.35 (95%CI: 1.33–4.16), *p* = 0.003; multiple complications (children *vs*. adults), odds ratio = 2.29 (95%CI: 0.85–6.13), *p* = 0.100; children as reference.

^*c*^ Other: bronchitis (n = 2), hemorrhagic rash (n = 1), febrile seizures (n = 1), skin desquamation on the face (n = 1), leukopenia (n = 1).

The majority of outbreak cases (94.2%; n = 210/223) have never been vaccinated against measles (Table 3). Only 3.6% (n = 8/223) received one dose of MMR/MMRV, including five children ≤5 years of age, still too young to complete the recommended immunization schedule, and two adults among the age-group 19–29 years. Only one subject with confirmed infection received two doses of vaccine (0.4%), while in four cases (1.8%) detailed vaccination data were unavailable.

**Table 3:** Laboratory confirmed measles cases by vaccination status and age-group, Sicily, March 2012—August 2017.

Number of confirmed measles cases, n (%)	Age-group (years)						
	Total	<1	1–4	5–9	10–18	19–29	≥30
**Vaccination status**	**n = 223**	**n = 11**	**n = 55**	**n = 21**	**n = 18**	**n = 62**	**n = 56**
**Unvaccinated**	210 (94.2)	11 (100.0)	50 (90.9)	20 (95.2)	16 (88.9)	57 (91.9)	56 (100.0)
**Vaccinated**	13 (5.8)	0	5 (9.1)	1 (4.8)	2 (11.1)	5 (8.1)	0
***One-dose***	*8 (3*.*6)*	*---*	*5 (9*.*1)*	*1 (4*.*8)*	*---*	*2 (3*.*2)*	*---*
***Two-dose***	*1 (0*.*4)*	*---*	*---*	*---*	*1 (5*.*6)*	*---*	*---*
***Unknown***	*4 (1*.*8)*	*---*	*---*	*---*	*1 (5*.*6)*	*3 (4*.*8)*	*---*

### Sequence and phylogenetic analysis

A total of 221 sequences of the partial N gene of wild-type MVs circulating in Sicily during the study period were obtained and phylogenetically analysed.

**Fig 1:**
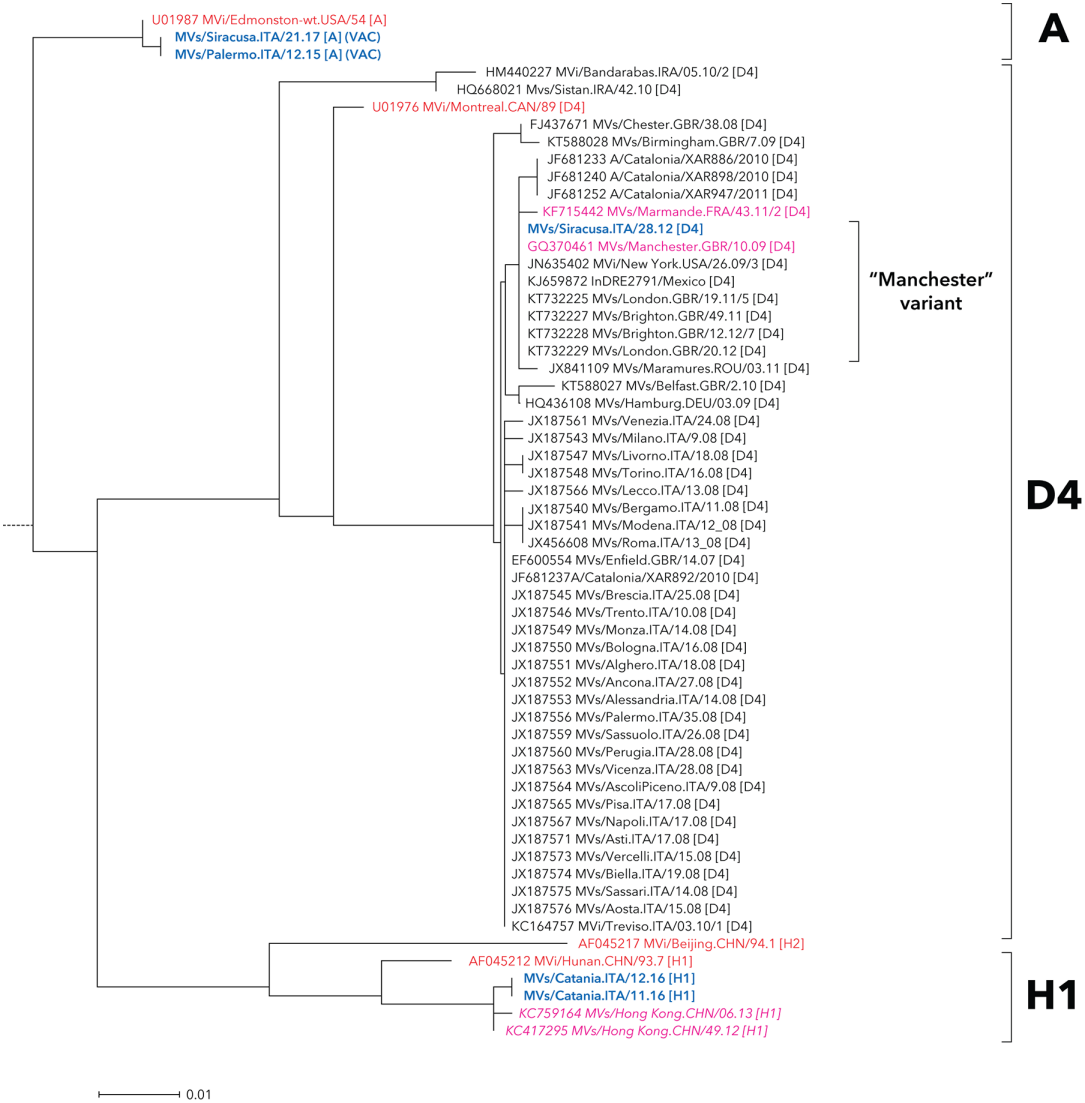
Neighbour-joining tree for measles nucleotide sequences beloging to genotypes A, D4 and H1. MV strains detected in Sicily are shown in bold and blue color. The reference and named strains recommended by WHO are included for comparison and indicated in red color (plain and italic, respectively), together with GenBank accession numbers. The tree was based on nucleotide sequences encoding the C-terminus of the MV N gene and rooted on the WHO reference strain MVs/Madrid.SPA/94 SSPE [F].

**Fig 2:**
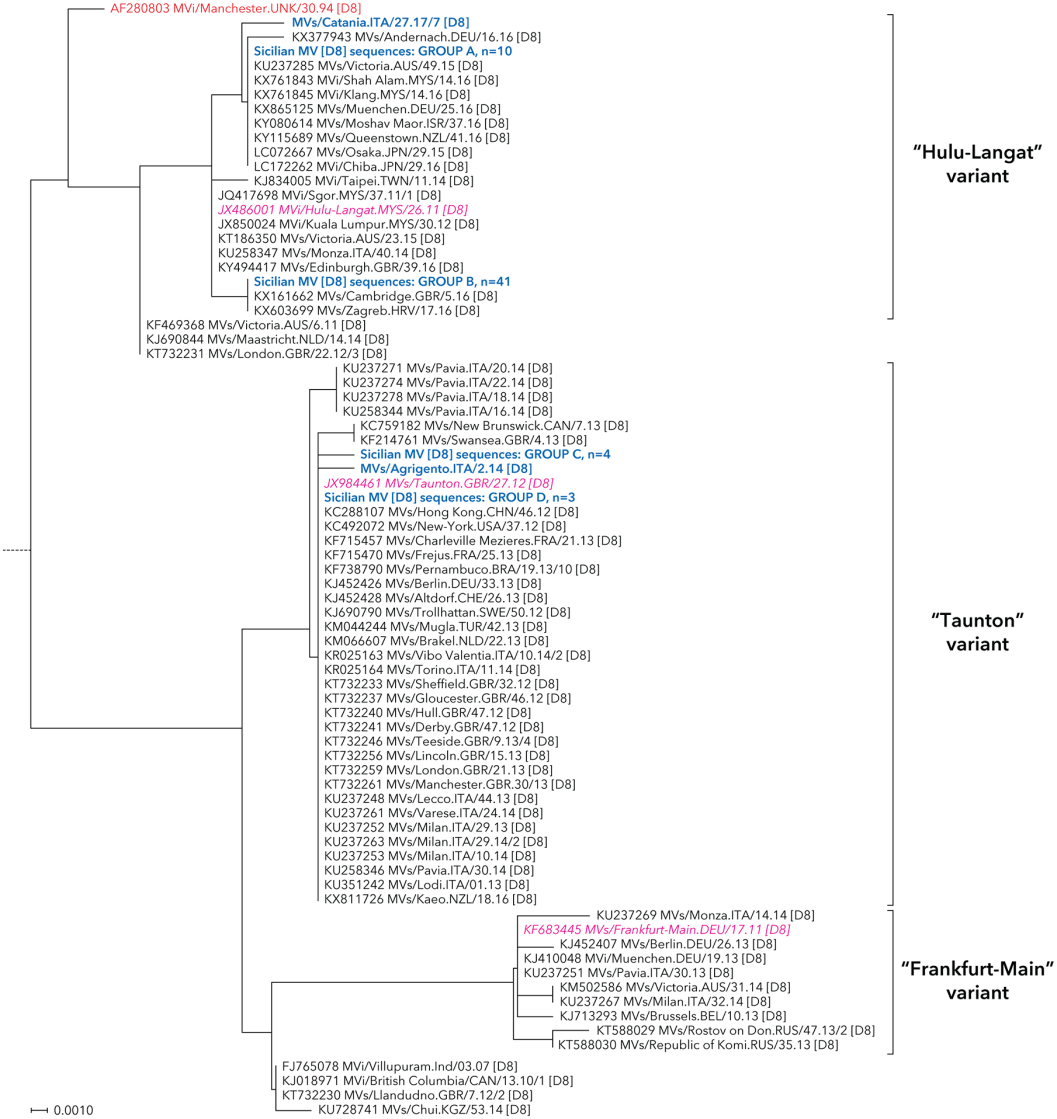
Neighbour-joining tree for measles nucleotide sequences beloging to genotypes D8. MV strains detected in Sicily are shown in bold and blue color. The reference and named strains recommended by WHO are included for comparison and indicated in red color (plain and italic, respectively), together with GenBank accession numbers. The tree was based on nucleotide sequences encoding the C-terminus of the MV N gene and rooted on the WHO reference strain MVs/Madrid.SPA/94 SSPE [F]. All relevant information on genotype D8 Sicilian MV sequences included into groups A-D are reported in S1 Table.

**Fig 3:**
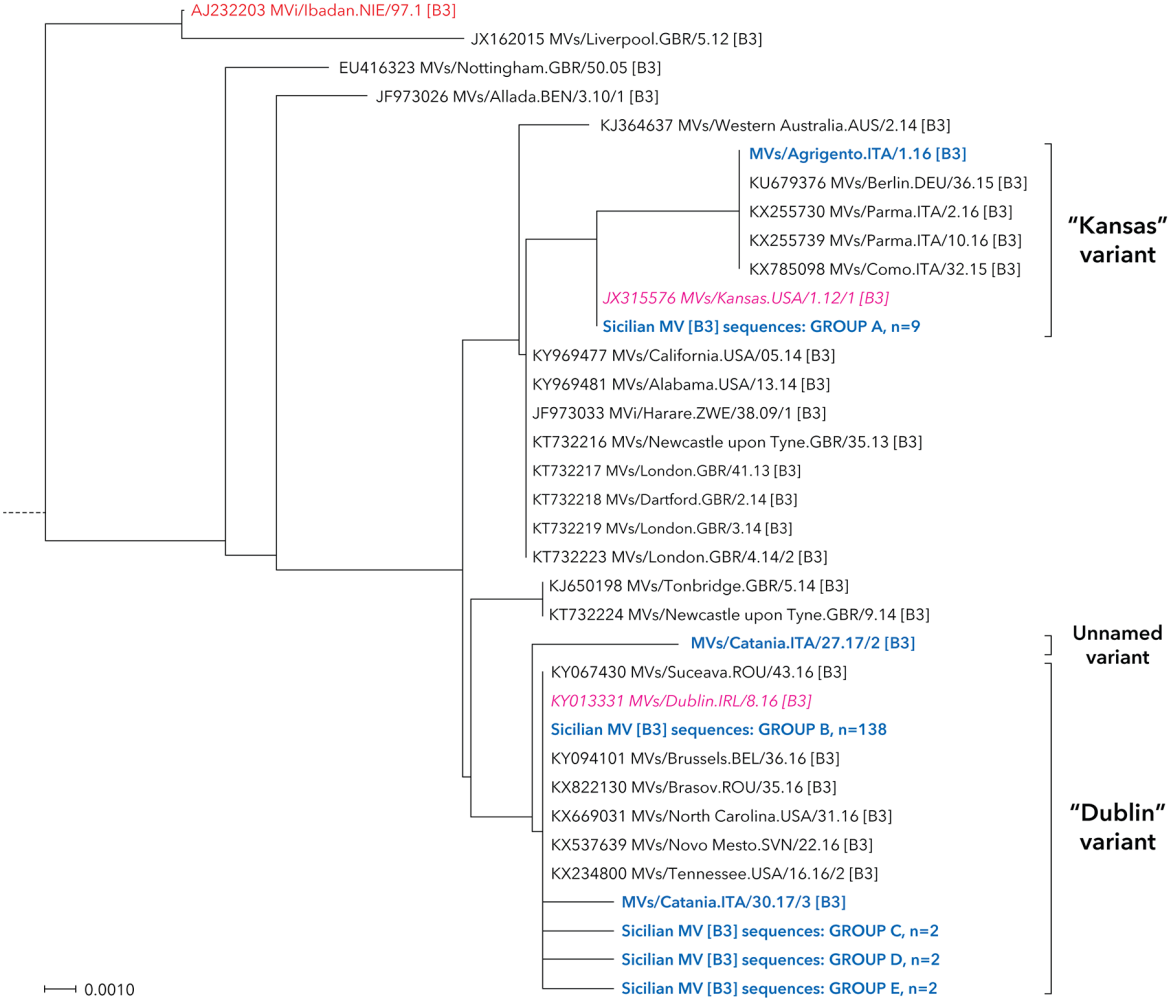
Neighbour-joining tree for measles nucleotide sequences beloging to genotypes A and B3. MV strains detected in Sicily are shown in bold and blue color. The reference and named strains recommended by WHO are included for comparison and indicated in red color (plain and italic, respectively), together with GenBank accession numbers. The tree was based on nucleotide sequences encoding the C-terminus of the MV N gene and rooted on the WHO reference strain MVs/Madrid.SPA/94 SSPE [F]. All relevant information on genotype D8 Sicilian MV sequences included into groups A-E are reported in S1 Table.

The nucleotide sequence analysis identified five different measles genotypes in Sicily: A, B3, D4, D8 and H1 (Figs 1–3), although MV sequences mainly fell in two major groups belonging to genotypes D8 (60 sequences; 27.1%) and B3 (156 sequences; 70.6%).

Over the study period, only one strain of genotype D4 was found (MVs/Siracusa.ITA/23.12), it belonged to the “Manchester” variant (MVs/Manchester.GBR/10/09) (Fig 1). H1 genotype measles sequences were identified in two adult subjects; one of them was infected abroad during a work period spent in China, while the other infection was locally transmitted by this subject to his partner.

Additionally, two vaccine-derived genotype A viruses were reported in infants after receiving their first dose of measles vaccine.

As depicted in Fig 2, the phylogenetic analysis of Sicilian MV sequences evidenced two main different clusters within the D8 genotype, closely related to the “Taunton” (MVs/Taunton.GBR/27.12) and the “Hulu-Langat” (MVi/Hulu-Langat.MYS/26.11) variants.

In total, 13.3% (n = 8/60) of D8 genotype measles strains circulating between October 2013 and February 2014 in the South-Eastern area of the region belonged to the “Taunton” variant.

Within this group, three Sicilian sequences resulted 100% identical to the WHO named strain and other MVs reported in different Italian regions in that time period. Besides, a single viral strain (MVs/Agrigento.ITA/2.14) and a small cluster of four identical sequences, two of which (MVs/Ragusa.ITA/42.13 and MVs/Ragusa.ITA/44.13/1) identified in children belonging to the same family, had no similarities in the BLAST database.

The “Hulu-Langat” was the most representative variant in Sicily (85.0%; n = 51/60). It firstly characterized an outbreak of measles that began in the westernmost part of Sicily in April 2016 and then progressively spread to the Central and Eastern part of the region. Afterwards, the D8 genotype was very rarely identified in our area and the molecular layout of circulating MVs substantially switched to the B3 genotype (Fig 3).

This genotype initially appeared in a sporadic measles case imported from the Northern Italy (MVs/Agrigento.ITA/1.16), whose sequence belonged to the “Kansas” (MVs/Kansas.USA/1.12/1) variant and strictly related to MVs identified in northern regions of Italy during the same period (Fig 3).

After that, in early 2017, the B3 genotype was consistently introduced in Sicily, being primarily represented by MVs identical to the “Kansas” WHO named strain within the Northwestern area and then rapidly spreading toward the Eastern part of the region. Finally, the “Dublin” (MVs/Dublin.IRL/8.16) named strain circulated in Sicily, clearly delineated the current measles outbreak of endemic cases and, as of 31 August 2017, 145 MVs have been identified; almost all (95.2%; n = 138/145) were 100% identical to this WHO named strain, while a unique sequence (MVs/Catania.ITA/27.17/2) fell outside the main cluster (unnamed variant).

**Fig 4:**
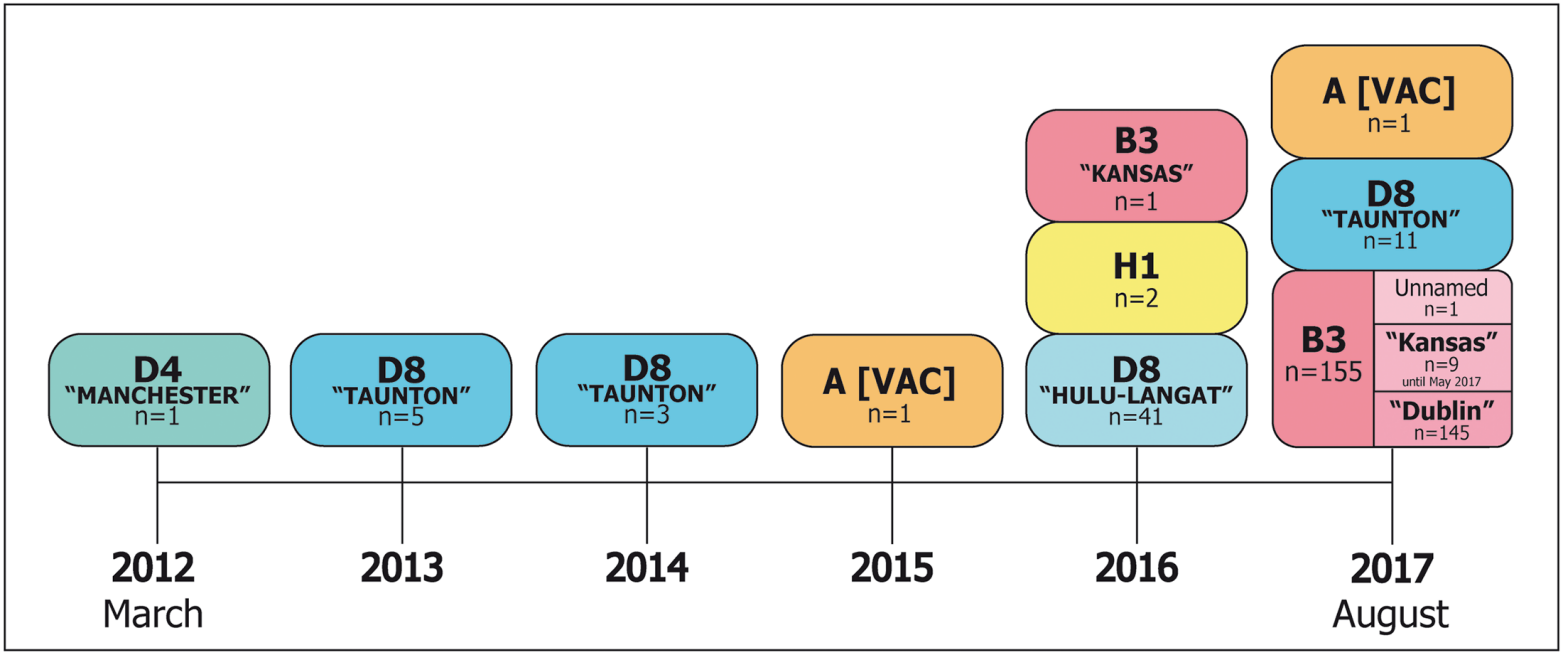
Timeline of measles genotypes and variants identified in Sicily during the surveillance period March 2012—August 2017.

In summary, Fig 4 illustrates the temporal distribution of wild-type MVs genotypes and relative genetic variants identified in Sicily during the study period.

## Discussion

The “Global Measles and Rubella Strategic Plan: 2012–2020” of the WHO [1] aimed to achieve the elimination of measles in Europe by 2015. To date, according to latest ECDC surveillance data on measles [20], only ten European Member States (EU/EEA) have reached the goal “reported zero cases”, whereas measles continues to spread across Europe because of suboptimal vaccination coverage in many EU/EEA countries. More than 17,000 cases of measles were notified in Europe from 1 June 2016 to end July 2017 and some of most populated countries, such as Italy, doubled the number of reported cases in January-February 2016.

Recently, the largest measles outbreak in Europe took place in Italy [20] affecting subjects with a median age of 27 years; 43% of confirmed cases were admitted into hospital [8].

In our setting, the proportion of measles cases reported by hospital wards was unexpectedly high, although this was not necessarily related to the presence of clinical complications. In this regard, however, it must be stressed that hospitalized patients were significantly younger than the cases in the community [21–23], most of them were children aged ≤5 years, and this may have potentially reflected a justifiable high-level attention in respect to this population group.

Moreover, in Northern Italy, Berti et al [22] evaluated the hospitalization rates in the period 2010–2014 and reported an increasing trend in young adults. This suggests an increase in susceptibility to measles in this subpopulation, which may consequently be considered an emergent priority in the national vaccination strategies, especially in countries where measles is still endemic. Additionally, our findings underlined that the majority of clinical complications, either single or multiple, occurred in adults and, according to others [8,11] the most frequent ones were diarrhoea, keratoconjuntivitis, pneumonia, and laryngotracheobronchitis.

In this work, the genetic characteristics of MVs circulating in Sicily over the last quinquennium were also analyzed.

The results highlight the circulation of different measles genotypes in our geographic area, variation in the molecular dynamics occurred year-by-year, characterizing different local outbreaks.

Two sporadic cases were associated to the genotype H1, 100% equal to each other, showing the highest similarity to MVs circulating between 2012 and 2013 in Hong Kong, where this genotype is still endemic [24,25]. Epidemiological data confirmed the phylogenetic relationships, being primarily derived from a subject returning from a work period in that area and then locally transmitted to his partner. Notably, no other H1 infections were notified thereafter.

Moreover, two detections of the genotype A were found in Sicily; these occurred in little children after the scheduled first dose of MMR vaccination, one of which had a concurrent influenza virus infection [26].

During the study period, a limited circulation of D4 genotype was documented in our region. This variant was only identified at the beginning of the surveillance period, when it appeared endemic at the national level [11,27]. After that, it was no longer observed because of an interruption of local circulation, being completely replaced by D8.

Within this latter MV genotype, two main sequence clusters were clearly identified. The first one grouped the measles strains circulating in Sicily between 2013 and 2014, these referred to the “Taunton” variant as also reported during the same period in Northern Italy [10,11].

On the other hand, a second group of sequences showed similarities with the “Hulu-Langat” variant and uniformely characterized a large measles outbreak firstly occurred in the Western Sicily in the second half of 2016 and then spreading in the neighbouring area of Palermo.

The 100% identity of MV sequences with the “Hulu-Langat” variant, together with the information gathered during the epidemiological investigations, suggested the persistence of a unique chain of transmission of this D8 variant during at least a 6-month period.

In Sicily, the last notification of measles cases belonging to the D8 genotype occurred about six months later in the Eastern part of the region, showing viral nucleotide sequences never reported before in Italy and substantially dissimilar from the other strains previously detected in our area.

Then, in the early 2016, a transition from genotype D8 to B3 was observed in Sicily. The introduction of this latter genotype chronologically correlated to a measles case imported from a Northern region of Italy, where the circulation of this viral variant began a couple of years before [11], and that was identical to a B3 variant spread from Lombardy to Emilia Romagna among the Roma/Sinti population and healthcare workers [28].

The B3 genotype was also largely documented in other European countries [23,29,30], being globally the third most commonly reported measles variant in the MeaNS/WHO database during the last 12-month period, and confirming the currently changing molecular epidemiology of the virus.

In this regard, interestingly, Ackley and colleagues [31] recently found a higher transmissibility of the B3 genotype, compared with all measles genotypes combined, suggesting a potential role of this variant in sustaining larger outbreaks with stronger public health impact.

On the whole, considering both epidemiological and molecular data from this and other reports focused on measles surveillance, some issues deserve attention.

First, our findings confirm that measles still represents a common childhood illness and this is even more evident in low-income countries [32] where it remains one of the leading causes of vaccine-preventable deaths in children <5 years [4].

Despite the implementation of measles universal immunization programmes, several industrialized countries achieved suboptimal MMR/MMRV vaccination coverages, as consequence of antivaccination movements arising from a now completely discredited claim of a link between MMR/MMRV vaccine and autism [33], and that have represented a major reason for the Italian government to recently introduce mandatory vaccination against some common disease, among which measles, for all children aged under 16 [34].

Low levels of immunization coverage prevented the complete interruption of viral spread among young children, thus experiencing variation in age-distribution of measles susceptibility toward adolescents and young adults, as observed in our setting, which consequently may represent the natural target of supplementary immunization activities.

Second, our results add evidence to the wider literature on the occurrence of measles predominantly among unvaccinated individuals [8,11,23,28,35]. This confirms that immunization against measles, with the current live-attenuated vaccine, represents the most effective preventive measure to control the spread of the disease in the general population and also providing good protection irrespective the remarkable genetic variability of circulating MVs [36].

Notwithstanding, other authors reported that some genotypes of MVs may exhibit different antigenicity [37,38] and it has been hypothesized that this could potentially lead to less efficiency of the current vaccine [39] or cause genotypic-specific escape to neutralization [40–42].

Fatemi Nasab and colleagues [43] reported about two-thirds lower geometric mean neutralizing antibody titers toward genotype B3, when compared to serum concentration levels against different genotypes such as D4, H1 and A and, notably, it has been demonstrated a higher pathogenicity of genotype B3 than other genotypes in a macaque animal model [44].

In conclusion, this work drawn attention to the changing scenario in measles epidemiology, confirming the increasing age of infected subjects, which could be more prone to hospitalization and severe complications. This adds evidence to an urgent need of updating the vaccination schedule against measles, implementing catch-up campaigns for children who missed one or all MMR/MMRV doses, as well as for susceptible population groups at any age.

Different temporal introductions of MVs variants were observed in Sicily, evidencing a clear variation in the molecular epidemiology of circulating measles strains during the entire review period, and pointed out the current predominance of the genotype B3.

The results presented here highlighted the importance of continuous measles surveillance and genotyping in order to support the epidemiological investigations in identifying outbreaks of autochthonous cases, to understand the molecular dynamics of MVs, and to investigate potential differences in immune response, in order to eventually adapt the development of successfull vaccine formulations.

## Supporting information

S1 Fig Number of confirmed measles cases documented during the period March 2012—August 2017 (n = 223). Geographic distribution by Sicilian province.
(TIF)

S1 Table Relevant information on Sicilian MV sequences included into phylogenetic trees. (PDF)

## Abbreviations

EU: European Union
LHA: Local Health Authority
MMR: Measles-Mumps-Rubella
MV: Measles virus
PNEMoRc: National Plan for the Elimination of Measles and Congenital Rubella
WHO: World Health Organization
