# Supplementary figures and images for: Case-based surveillance of measles in Sicily during 2012-2017: The changing molecular epidemiology and implications for vaccine strategies
Source: PLoS One. 2018 Apr 4;13(4):e0195256. doi: 10.1371/journal.pone.0195256 (PMC5884552; doi:10.1371/journal.pone.0195256)

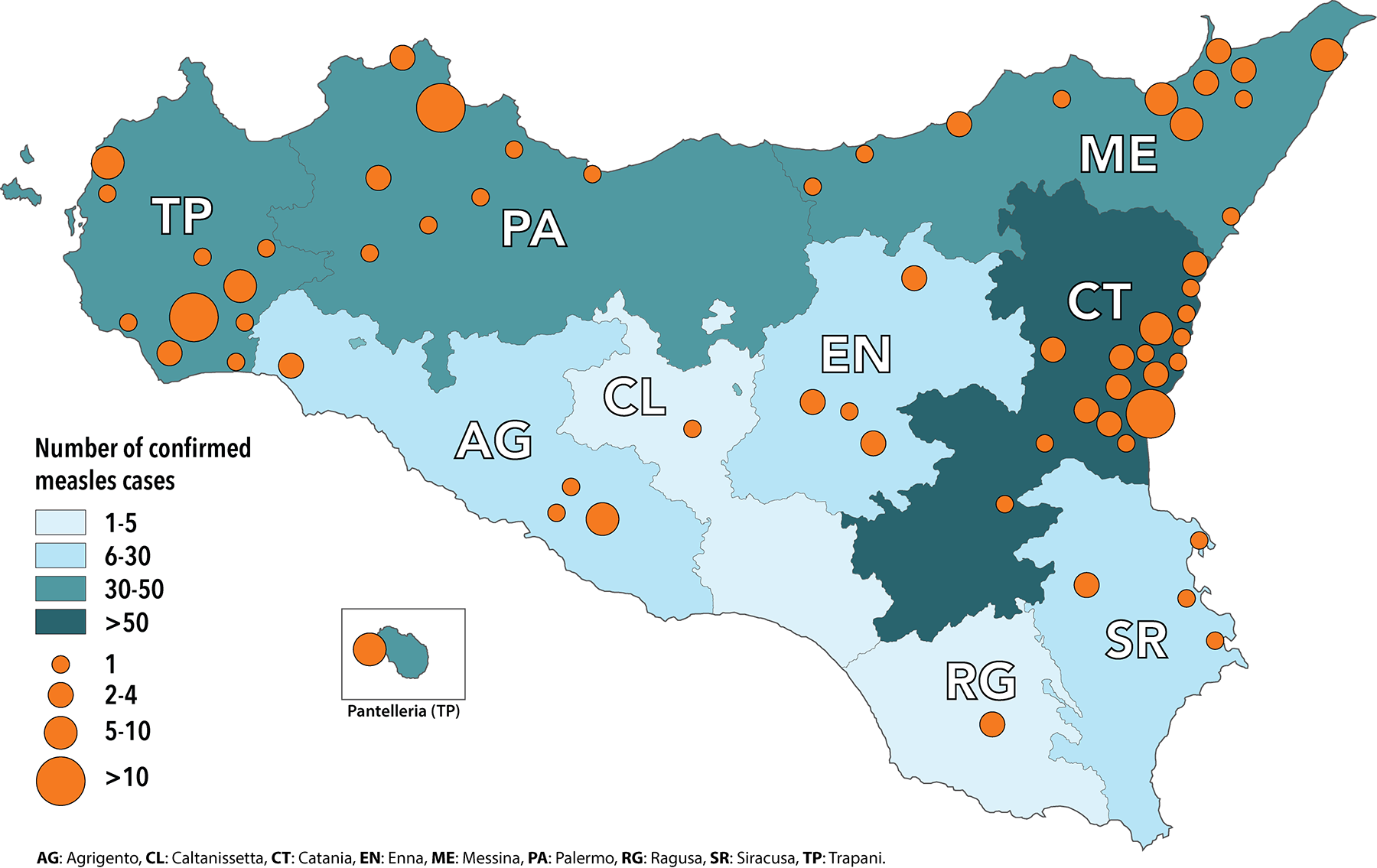

Supplement: S1 Fig — Geographic distribution by Sicilian province. (TIF) [file pone.0195256.s001.tif]
