# Supplementary material for: Case-based surveillance of measles in Sicily during 2012-2017: The changing molecular epidemiology and implications for vaccine strategies
Source: PLoS One. 2018 Apr 4;13(4):e0195256. doi: 10.1371/journal.pone.0195256 (PMC5884552; doi:10.1371/journal.pone.0195256)
Supplement: S1 Table — (PDF) [file pone.0195256.s002.pdf]

| NUMBER | STRAIN DESIGNATION | AGE (YEAR) | GENDER | HEALTH SETTING | BIOLOGICAL SAMPLE | GENOTYPE | TREE GROUP | VACCINE HISTORY | GENBANK ACCESSION NUMBER |
|---|---|---|---|---|---|---|---|---|---|
| **1** | MVs/Palermo.ITA/12.15 [A] (VAC) | 1 | M | Hospital | OS+U | A | | Yes | KR262162 |
| **2** | MVs/Siracusa.ITA/21.17 [A] (VAC) | 1 | F | Community | OS+U | A | | Yes | Submitted |
| **3** | MVs/Agrigento.ITA/1.16 [B3] | 1 | F | Hospital | OS+U | B3 | | No | KY801709 |
| **4** | MVs/Palermo.ITA/4.17/1 [B3] | 10M | M | Community | U | B3 | GROUP B | No | KY801744 |
| **5** | MVs/Palermo.ITA/4.17/2 [B3] | 27 | F | Hospital | OS+U | B3 | GROUP B | No | KY801745 |
| **6** | MVs/Messina.ITA/5.17 [B3] | 41 | M | Community | U | B3 | GROUP B | No | KY801723 |
| **7** | MVs/Messina.ITA/6.17 [B3] | 4 | F | Community | U | B3 | GROUP B | No | KY801724 |
| **8** | MVs/Messina.ITA/7.17/1 [B3] | 7 | F | Hospital | U | B3 | GROUP B | No | KY801725 |
| **9** | MVs/Messina.ITA/7.17/2 [B3] | 1 | M | Hospital | OS+U | B3 | GROUP B | No | KY801726 |
| **10** | MVs/Palermo.ITA/7.17 [B3] | 8 | M | Hospital | OS+U | B3 | GROUP B | No | KY801746 |
| **11** | MVs/Messina.ITA/8.17/2 [B3] | 2 | M | Community | U | B3 | GROUP B | No | KY801728 |
| **12** | MVs/Messina.ITA/8.17/3 [B3] | 8 | F | Community | U | B3 | GROUP B | No | KY801729 |
| **13** | MVs/Messina.ITA/8.17/1 [B3] | 5 | F | Community | U | B3 | GROUP B | No | KY801727 |
| **14** | MVs/Messina.ITA/9.17 [B3] | 25 | M | Hospital | U | B3 | GROUP B | No | KY801730 |
| **15** | MVs/Messina.ITA/11.17/1 [B3] | 24 | M | Hospital | U | B3 | GROUP B | No | KY801720 |
| **16** | MVs/Palermo.ITA/10.17 [B3] | 1 | F | Hospital | U | B3 | GROUP B | No | KY801731 |
| **17** | MVs/Messina.ITA/11.17/3 [B3] | 19 | F | Hospital | U | B3 | GROUP B | No | KY801722 |
| **18** | MVs/Messina.ITA/11.17/2 [B3] | 1 | M | Hospital | U | B3 | GROUP B | No | KY801721 |
| **19** | MVs/Palermo.ITA/12.17 [B3] | 1 | M | Hospital | OS+U | B3 | GROUP B | No | KY801732 |
| **20** | MVs/Messina.ITA/12.17/1 [B3] | 27 | F | Hospital | OS | B3 | GROUP B | No | Submitted |
| **21** | MVs/Messina.ITA/12.17/2 [B3] | 27 | M | Hospital | U | B3 | GROUP B | No | Submitted |
| **22** | MVs/Messina.ITA/12.17/3 [B3] | 1 | M | Hospital | U | B3 | GROUP B | No | Submitted |
| **23** | MVs/Messina.ITA/12.17/4 [B3] | 2 | F | Hospital | U | B3 | GROUP B | No | Submitted |
| **24** | MVs/Enna.ITA/12.17 [B3] | 28 | F | Hospital | U | B3 | GROUP B | No | Submitted |
| **25** | MVs/Messina.ITA/12.17/5 [B3] | 40 | F | Community | U | B3 | GROUP B | No | Submitted |
| **26** | MVs/Messina.ITA/12.17/6 [B3] | 26 | M | Hospital | U | B3 | GROUP B | No | Submitted |
| **27** | MVs/Enna.ITA/13.17/2 [B3] | 39 | M | Hospital | U | B3 | GROUP B | No | Submitted |
| **28** | MVs/Messina.ITA/14.17/1 [B3] | 1 | F | Hospital | U | B3 | GROUP B | No | Submitted |
| **29** | MVs/Messina.ITA/14.17/2 [B3] | 27 | F | Community | U | B3 | GROUP B | No | Submitted |
| **30** | MVs/Messina.ITA/14.17/3 [B3] | 5 | F | Community | U | B3 | GROUP B | No | Submitted |
| **31** | MVs/Messina.ITA/14.17/5 [B3] | 4 | F | Hospital | U | B3 | GROUP B | No | Submitted |
| **32** | MVs/Messina.ITA/14.17/4 [B3] | 3 | M | Community | U | B3 | GROUP B | No | Submitted |
| **33** | MVs/Messina.ITA/14.17/6 [B3] | 3 | F | Community | U | B3 | GROUP B | No | Submitted |
| **34** | MVs/Messina.ITA/14.17/7 [B3] | 35 | M | Hospital | U | B3 | GROUP B | No | Submitted |
| **35** | MVs/Enna.ITA/15.17 [B3] | 24 | M | Hospital | OS+U | B3 | GROUP B | No | Submitted |
| **36** | MVs/Palermo.ITA/15.17 [B3] | 1 | F | Hospital | OS+U | B3 | GROUP A | No | Submitted |
| **37** | MVs/Messina.ITA/15.17/1 [B3] | 1 | F | Hospital | U | B3 | GROUP B | No | Submitted |
| **38** | MVs/Messina.ITA/15.17/2 [B3] | 1 | M | Hospital | OS+U | B3 | GROUP B | Yes | Submitted |

| | | | | | | | | | |
|---|---|---|---|---|---|---|---|---|---|
| **39** | MVs/Palermo.ITA/16.17 [B3] | 1 | F | Hospital | OS+U | B3 | GROUP A | No | Submitted |
| **40** | MVs/Messina.ITA/17.17/1 [B3] | 1 | M | Hospital | U | B3 | GROUP B | Yes | Submitted |
| **41** | MVs/Messina.ITA/17.17/2 [B3] | 5 | M | Community | U | B3 | GROUP B | No | Submitted |
| **42** | MVs/Palermo.ITA/17.17 [B3] | 5M | M | Hospital | OS+U | B3 | GROUP A | No | Submitted |
| **43** | MVs/Palermo.ITA/18.17/1 [B3] | 25 | F | Hospital | U | B3 | GROUP A | No | Submitted |
| **44** | MVs/Palermo.ITA/18.17/2 [B3] | 3 | M | Hospital | OS+U | B3 | GROUP A | No | Submitted |
| **45** | MVs/Palermo.ITA/18.17/3 [B3] | 34 | M | Community | OS+U | B3 | GROUP A | No | Submitted |
| **46** | MVs/Palermo.ITA/21.17/2 [B3] | 21 | M | Hospital | U | B3 | GROUP A | No | Submitted |
| **47** | MVs/Messina.ITA/19.17/1 [B3] | 42 | M | Community | U | B3 | GROUP B | No | Submitted |
| **48** | MVs/Messina.ITA/19.17/2 [B3] | 29 | M | Hospital | OS+U | B3 | GROUP B | No | Submitted |
| **49** | MVs/Catania.ITA/19.17 [B3] | 13 | M | Hospital | U | B3 | GROUP B | No | Submitted |
| **50** | MVs/Messina.ITA/20.17/1 [B3] | 1 | F | Hospital | U | B3 | GROUP B | No | Submitted |
| **51** | MVs/Catania.ITA/20.17 [B3] | 4 | M | Hospital | OS+U | B3 | GROUP B | No | Submitted |
| **52** | MVs/Messina.ITA/20.17/2 [B3] | 26 | M | Hospital | U | B3 | GROUP B | No | Submitted |
| **53** | MVs/Palermo.ITA/20.17/2 [B3] | 9 | F | Hospital | OS+U | B3 | GROUP A | No | Submitted |
| **54** | MVs/Palermo.ITA/20.17/1 [B3] | 30 | M | Hospital | OS+U | B3 | GROUP A | No | Submitted |
| **55** | MVs/Catania.ITA/21.17/1 [B3] | 6 | M | Hospital | OS+U | B3 | GROUP B | No | Submitted |
| **56** | MVs/Messina.ITA/21.17 [B3] | 11 | F | Hospital | U | B3 | GROUP B | No | Submitted |
| **57** | MVs/Catania.ITA/21.17/2 [B3] | 24 | F | Hospital | OS+U | B3 | GROUP B | No | Submitted |
| **58** | MVs/Siracusa.ITA/22.17 [B3] | 27 | F | Community | U | B3 | GROUP B | No | Submitted |
| **59** | MVs/Catania.ITA/22.17/1 [B3] | 36 | M | Hospital | OS+U | B3 | GROUP B | No | Submitted |
| **60** | MVs/Catania.ITA/22.17/2 [B3] | 12 | M | Hospital | OS+U | B3 | GROUP B | No | Submitted |
| **61** | MVs/Catania.ITA/23.17/2 [B3] | 22 | F | Hospital | OS+U | B3 | GROUP B | No | Submitted |
| **62** | MVs/Catania.ITA/23.17/1 [B3] | 38 | M | Hospital | OS+U | B3 | GROUP B | No | Submitted |
| **63** | MVs/Messina.ITA/23.17 [B3] | 45 | M | Hospital | OS+U | B3 | GROUP B | No | Submitted |
| **64** | MVs/Catania.ITA/23.17/3 [B3] | 18 | M | Hospital | U | B3 | GROUP B | No | Submitted |
| **65** | MVs/Catania.ITA/24.17/4 [B3] | 27 | M | Hospital | U | B3 | GROUP B | Yes | Submitted |
| **66** | MVs/Catania.ITA/24.17/5 [B3] | 21 | M | Hospital | U | B3 | GROUP B | Yes | Submitted |
| **67** | MVs/Catania.ITA/23.17/6 [B3] | 17 | M | Hospital | U | B3 | GROUP B | Yes | Submitted |
| **68** | MVs/Catania.ITA/23.17/7 [B3] | 27 | M | Community | U | B3 | GROUP B | No | Submitted |
| **69** | MVs/Catania.ITA/24.17/1 [B3] | 27 | F | Community | OS+U | B3 | GROUP B | No | Submitted |
| **70** | MVs/Trapani.ITA/24.17 [B3] | 28 | F | Hospital | U | B3 | GROUP B | No | Submitted |
| **71** | MVs/Catania.ITA/24.17/3 [B3] | 33 | M | Hospital | U | B3 | GROUP B | No | Submitted |
| **72** | MVs/Catania.ITA/24.17/2 [B3] | 26 | F | Hospital | OS+U | B3 | GROUP B | No | Submitted |
| **73** | MVs/Messina.ITA/24.17 [B3] | 7 | F | Hospital | U | B3 | GROUP B | No | Submitted |
| **74** | MVs/Catania.ITA/25.17/2 [B3] | 2 | F | Hospital | OS+U | B3 | GROUP B | No | Submitted |
| **75** | MVs/Catania.ITA/25.17/3 [B3] | 2 | F | Hospital | OS+U | B3 | GROUP B | No | Submitted |
| **76** | MVs/Catania.ITA/25.17/1 [B3] | 37 | F | Hospital | OS+U | B3 | GROUP B | No | Submitted |
| **77** | MVs/Messina.ITA/25.17 [B3] | 28 | F | Hospital | OS+U | B3 | GROUP B | No | Submitted |
| **78** | MVs/Catania.ITA/25.17/4 [B3] | 33 | F | Hospital | U | B3 | GROUP B | No | Submitted |

| | | | | | | | | | |
|---|---|---|---|---|---|---|---|---|---|
| **79** | MVs/Catania.ITA/25.17/5 [B3] | 35 | F | Hospital | U | B3 | GROUP B | No | Submitted |
| **80** | MVs/Catania.ITA/25.17/6 [B3] | 23 | F | Hospital | OS | B3 | GROUP B | No | Submitted |
| **81** | MVs/Enna.ITA/25.17 [B3] | 34 | F | Hospital | U | B3 | GROUP B | No | Submitted |
| **82** | MVs/Catania.ITA/25.17/7 [B3] | 13 | F | Hospital | OS+U | B3 | GROUP B | No | Submitted |
| **83** | MVs/Catania.ITA/25.17/8 [B3] | 29 | F | Hospital | U | B3 | GROUP B | No | Submitted |
| **84** | MVs/Catania.ITA/25.17/9 [B3] | 1 | M | Hospital | OS+U | B3 | GROUP B | No | Submitted |
| **85** | MVs/Trapani.ITA/26.17/1 [B3] | 25 | M | Hospital | U | B3 | GROUP B | No | Submitted |
| **86** | MVs/Catania.ITA/26.17/1 [B3] | 45 | M | Community | U | B3 | GROUP B | No | Submitted |
| **87** | MVs/Trapani.ITA/26.17/3 [B3] | 42 | M | Hospital | U | B3 | GROUP B | No | Submitted |
| **88** | MVs/Catania.ITA/26.17/2 [B3] | 20 | F | Hospital | U | B3 | GROUP E | No | Submitted |
| **89** | MVs/Trapani.ITA/26.17/2 [B3] | 44 | F | Hospital | U | B3 | GROUP B | No | Submitted |
| **90** | MVs/Trapani.ITA/26.17/4 [B3] | 25 | F | Community | U | B3 | GROUP B | No | Submitted |
| **91** | MVs/Catania.ITA/26.17/3 [B3] | 11 | M | Hospital | OS+U | B3 | GROUP B | No | Submitted |
| **92** | MVs/Trapani.ITA/26.17/6 [B3] | 1 | F | Hospital | U | B3 | GROUP B | No | Submitted |
| **93** | MVs/Trapani.ITA/26.17/5 [B3] | 13 | M | Hospital | U | B3 | GROUP B | No | Submitted |
| **94** | MVs/Trapani.ITA/26.17/7 [B3] | 23 | M | Hospital | U | B3 | GROUP B | No | Submitted |
| **95** | MVs/Catania.ITA/27.17/1 [B3] | 1 | M | Hospital | OS+U | B3 | GROUP B | No | Submitted |
| **96** | MVs/Catania.ITA/27.17/6 [B3] | 26 | F | Hospital | OS+U | B3 | GROUP B | No | Submitted |
| **97** | MVs/Catania.ITA/27.17/2 [B3] | 23 | F | Hospital | OS+U | B3 | | No | Submitted |
| **98** | MVs/Catania.ITA/27.17/4 [B3] | 3 | F | Hospital | U | B3 | GROUP B | No | Submitted |
| **99** | MVs/Catania.ITA/27.17/3 [B3] | 14 | F | Hospital | OS+U | B3 | GROUP B | No | Submitted |
| **100** | MVs/Catania.ITA/27.17/8 [B3] | 36 | F | Hospital | OS+U | B3 | GROUP B | No | Submitted |
| **101** | MVs/Catania.ITA/27.17/9 [B3] | 33 | F | Hospital | U | B3 | GROUP B | No | Submitted |
| **102** | MVs/Catania.ITA/27.17/5 [B3] | 7 | M | Hospital | OS+U | B3 | GROUP B | No | Submitted |
| **103** | MVs/Palermo.ITA/27.17/1 [B3] | 3 | M | Hospital | OS+U | B3 | GROUP B | No | Submitted |
| **104** | MVs/Palermo.ITA/27.17/2 [B3] | 31 | M | Community | U | B3 | GROUP B | No | Submitted |
| **105** | MVs/Trapani.ITA/28.17 [B3] | 46 | M | Hospital | U | B3 | GROUP B | No | Submitted |
| **106** | MVs/Catania.ITA/28.17 [B3] | 50 | F | Hospital | OS+U | B3 | GROUP B | No | Submitted |
| **107** | MVs/Catania.ITA/29.17/1 [B3] | 66 | F | Hospital | OS+U | B3 | GROUP B | No | Submitted |
| **108** | MVs/Enna.ITA/29.17/2 [B3] | 27 | F | Hospital | OS+U | B3 | GROUP B | No | Submitted |
| **109** | MVs/Enna.ITA/29.17/1 [B3] | 30 | F | Hospital | OS+U | B3 | GROUP C | No | Submitted |
| **110** | MVs/Palermo.ITA/29.17/2 [B3] | 7 | M | Hospital | OS+U | B3 | GROUP B | No | Submitted |
| **111** | MVs/Palermo.ITA/29.17/1 [B3] | 4 | F | Hospital | OS+U | B3 | GROUP B | No | Submitted |
| **112** | MVs/Palermo.ITA/29.17/3 [B3] | 3 | M | Hospital | OS+U | B3 | GROUP B | No | Submitted |
| **113** | MVs/Catania.ITA/29.17/2 [B3] | 2 | F | Hospital | OS+U | B3 | GROUP B | No | Submitted |
| **114** | MVs/Palermo.ITA/29.17/4 [B3] | 1 | M | Hospital | OS+U | B3 | GROUP B | No | Submitted |
| **115** | MVs/Palermo.ITA/29.17/5 [B3] | 12 | M | Hospital | OS+U | B3 | GROUP B | No | Submitted |
| **116** | MVs/Trapani.ITA/29.17 [B3] | 40 | F | Hospital | U | B3 | GROUP B | No | Submitted |
| **117** | MVs/Catania.ITA/29.17/3 [B3] | 1 | M | Hospital | OS+U | B3 | GROUP B | No | Submitted |
| **118** | MVs/Palermo.ITA/29.17/6 [B3] | 6M | M | Hospital | OS+U | B3 | GROUP D | No | Submitted |

| | | | | | | | | | |
|---|---|---|---|---|---|---|---|---|---|
| **119** | MVs/Catania.ITA/29.17/4 [B3] | 2 | M | Hospital | OS+U | B3 | GROUP B | No | Submitted |
| **120** | MVs/Catania.ITA/30.17/1 [B3] | 33 | M | Hospital | OS+U | B3 | GROUP B | No | Submitted |
| **121** | MVs/Catania.ITA/30.17/2 [B3] | 1 | M | Hospital | OS+U | B3 | GROUP B | No | Submitted |
| **122** | MVs/Catania.ITA/30.17/3 [B3] | 40 | F | Hospital | OS+U | B3 | | No | Submitted |
| **123** | MVs/Palermo.ITA/30.17/1 [B3] | 1 | F | Hospital | OS+U | B3 | GROUP B | No | Submitted |
| **124** | MVs/Palermo.ITA/30.17/2 [B3] | 9M | F | Hospital | OS+U | B3 | GROUP B | No | Submitted |
| **125** | MVs/Catania.ITA/30.17/5 [B3] | 49 | F | Hospital | OS+U | B3 | GROUP B | No | Submitted |
| **126** | MVs/Catania.ITA/30.17/4 [B3] | 34 | M | Hospital | OS+U | B3 | GROUP B | No | Submitted |
| **127** | MVs/Trapani.ITA/30.17 [B3] | 26 | F | Hospital | U | B3 | GROUP B | No | Submitted |
| **128** | MVs/Catania.ITA/30.17/6 [B3] | 32 | M | Hospital | OS+U | B3 | GROUP B | No | Submitted |
| **129** | MVs/Palermo.ITA/30.17/3 [B3] | 1 | F | Hospital | OS+U | B3 | GROUP B | No | Submitted |
| **130** | MVs/Palermo.ITA/30.17/4 [B3] | 1 | M | Hospital | OS+U | B3 | GROUP B | No | Submitted |
| **131** | MVs/Enna.ITA/31.17 [B3] | 30 | F | Hospital | OS+U | B3 | GROUP B | No | Submitted |
| **132** | MVs/Palermo.ITA/31.17/2 [B3] | 5 | F | Hospital | OS+U | B3 | GROUP B | No | Submitted |
| **133** | MVs/Palermo.ITA/31.17/1 [B3] | 1 | F | Hospital | OS+U | B3 | GROUP B | No | Submitted |
| **134** | MVs/Catania.ITA/31.17/2 [B3] | 30 | M | Hospital | U | B3 | GROUP B | No | Submitted |
| **135** | MVs/Catania.ITA/31.17/3 [B3] | 30 | M | Hospital | OS+U | B3 | GROUP B | No | Submitted |
| **136** | MVs/Catania.ITA/31.17/1 [B3] | 1 | M | Hospital | OS+U | B3 | GROUP B | No | Submitted |
| **137** | MVs/Catania.ITA/31.17/4 [B3] | 3 | F | Hospital | OS+U | B3 | GROUP E | Yes | Submitted |
| **138** | MVs/Trapani.ITA/31.17/1 [B3] | 34 | F | Community | OS+U | B3 | GROUP B | No | Submitted |
| **139** | MVs/Catania.ITA/31.17/5 [B3] | 1 | F | Hospital | OS+U | B3 | GROUP B | No | Submitted |
| **140** | MVs/Catania.ITA/31.17/6 [B3] | 22 | F | Hospital | OS+U | B3 | GROUP B | No | Submitted |
| **141** | MVs/Palermo.ITA/31.17/3 [B3] | 12 | M | Hospital | OS+U | B3 | GROUP B | No | Submitted |
| **142** | MVs/Trapani.ITA/31.17/2 [B3] | 39 | M | Community | OS+U | B3 | GROUP B | No | Submitted |
| **143** | MVs/Catania.ITA/31.17/7 [B3] | 6 | F | Hospital | OS+U | B3 | GROUP B | Yes | Submitted |
| **144** | MVs/Messina.ITA/32.17 [B3] | 2 | M | Hospital | U | B3 | GROUP B | No | Submitted |
| **145** | MVs/Palermo.ITA/32.17/1 [B3] | 6 | F | Hospital | OS+U | B3 | GROUP B | No | Submitted |
| **146** | MVs/Catania.ITA/32.17 [B3] | 34 | M | Community | U | B3 | GROUP B | No | Submitted |
| **147** | MVs/Palermo.ITA/32.17/2 [B3] | 1 | M | Hospital | U | B3 | GROUP B | No | Submitted |
| **148** | MVs/Enna.ITA/32.17 [B3] | 28 | M | Hospital | OS+U | B3 | GROUP C | No | Submitted |
| **149** | MVs/Catania.ITA/33.17/1 [B3] | 10M | F | Hospital | OS | B3 | GROUP B | No | Submitted |
| **150** | MVs/Palermo.ITA/33.17 [B3] | 26 | M | Hospital | OS+U | B3 | GROUP D | No | Submitted |
| **151** | MVs/Catania.ITA/33.17/2 [B3] | 5 | F | Hospital | OS+U | B3 | GROUP B | No | Submitted |
| **152** | MVs/Catania.ITA/34.17/1 [B3] | 25 | F | Hospital | OS+U | B3 | GROUP B | No | Submitted |
| **153** | MVs/Messina.ITA/34.17/1 [B3] | 25 | M | Hospital | U | B3 | GROUP B | No | Submitted |
| **154** | MVs/Catania.ITA/34.17/2 [B3] | 1 | F | Hospital | OS+U | B3 | GROUP B | No | Submitted |
| **155** | MVs/Messina.ITA/34.17/2 [B3] | 48 | M | Hospital | U | B3 | GROUP B | No | Submitted |
| **156** | MVs/Palermo.ITA/34.17 [B3] | 28 | F | Hospital | OS+U | B3 | GROUP B | No | Submitted |
| **157** | MVs/Palermo.ITA/35.17 [B3] | 30 | M | Hospital | OS+U | B3 | GROUP B | No | Submitted |
| **158** | MVs/Trapani.ITA/35.17 [B3] | 25 | M | Community | OS+U | B3 | GROUP B | Yes | Submitted |

| | | | | | | | | | |
|---|---|---|---|---|---|---|---|---|---|
| **159** | MVs/Siracusa.ITA/28.12 [D4] | 14 | M | Community | OS+U | D4 | | No | KY801750 |
| **160** | MVs/Siracusa.ITA/37.13/1 [D8] | 12 | M | Community | OS | D8 | GROUP C | No | KY801751 |
| **161** | MVs/Siracusa.ITA/37.13/2 [D8] | 11M | F | Community | OS | D8 | GROUP C | No | KY801752 |
| **162** | MVs/Ragusa.ITA/42.13 [D8] | 23 | F | Hospital | U | D8 | GROUP C | No | KY801747 |
| **163** | MVs/Ragusa.ITA/44.13/1 [D8] | 26 | M | Hospital | U | D8 | GROUP C | No | KY801748 |
| **164** | MVs/Ragusa.ITA/44.13/2 [D8] | 47 | F | Hospital | U | D8 | GROUP D | No | KY801749 |
| **165** | MVs/Agrigento.ITA/2.14 [D8] | 21 | M | Community | OS+U | D8 | | No | KY801710 |
| **166** | MVs/Catania.ITA/4.14 [D8] | 10 | M | Community | OS+U | D8 | GROUP D | No | KY801717 |
| **167** | MVs/Catania.ITA/9.14 [D8] | 4 | M | Community | OS+U | D8 | GROUP D | No | KY801719 |
| **168** | MVs/Trapani.ITA/37.16 [D8] | 18 | F | Community | U | D8 | GROUP B | No | KY801774 |
| **169** | MVs/Trapani.ITA/15.16 [D8] | 26 | M | Community | U | D8 | GROUP B | No | KY801753 |
| **170** | MVs/Trapani.ITA/16.16/2 [D8] | 22 | M | Community | U | D8 | GROUP B | No | KY801755 |
| **171** | MVs/Trapani.ITA/17.16/1 [D8] | 26 | M | Community | OS+U | D8 | GROUP B | No | KY801758 |
| **172** | MVs/Trapani.ITA/16.16/3 [D8] | 1 | M | Hospital | OS+U | D8 | GROUP B | No | KY801756 |
| **173** | MVs/Trapani.ITA/16.16/4 [D8] | 45 | F | Community | U | D8 | GROUP B | No | KY801757 |
| **174** | MVs/Trapani.ITA/17.16/3 [D8] | 41 | M | Community | U | D8 | GROUP B | No | KY801760 |
| **175** | MVs/Trapani.ITA/17.16/2 [D8] | 2 | F | Hospital | OS+U | D8 | GROUP B | No | KY801759 |
| **176** | MVs/Trapani.ITA/18.16/1 [D8] | 2 | F | Hospital | OS+U | D8 | GROUP B | No | KY801761 |
| **177** | MVs/Trapani.ITA/16.16/1 [D8] | 5 | M | Hospital | OS+U | D8 | GROUP B | No | KY801754 |
| **178** | MVs/Trapani.ITA/18.16/2 [D8] | 33 | F | Community | OS+U | D8 | GROUP B | No | KY801762 |
| **179** | MVs/Trapani.ITA/18.16/3 [D8] | 10M | M | Hospital | OS+U | D8 | GROUP B | No | KY801763 |
| **180** | MVs/Trapani.ITA/19.16/2 [D8] | 45 | M | Hospital | OS+U | D8 | GROUP B | No | KY801765 |
| **181** | MVs/Trapani.ITA/19.16/3 [D8] | 23 | F | Community | OS+U | D8 | GROUP B | No | KY801766 |
| **182** | MVs/Trapani.ITA/19.16/1 [D8] | 26 | F | Community | U | D8 | GROUP B | No | KY801764 |
| **183** | MVs/Trapani.ITA/20.16/2 [D8] | 25 | M | Community | OS+U | D8 | GROUP B | No | KY801768 |
| **184** | MVs/Palermo.ITA/21.16/1 [D8] | 9 | F | Hospital | OS+U | D8 | GROUP B | No | KY801733 |
| **185** | MVs/Catania.ITA/20.16 [D8] | 3 | F | Community | OS+U | D8 | GROUP B | No | KY801716 |
| **186** | MVs/Trapani.ITA/20.16/1 [D8] | 29 | M | Community | OS+U | D8 | GROUP B | No | KY801767 |
| **187** | MVs/Trapani.ITA/21.16 [D8] | 23 | F | Community | OS+U | D8 | GROUP B | No | KY801769 |
| **188** | MVs/Trapani.ITA/22.16 [D8] | 24 | M | Community | U | D8 | GROUP B | Yes | KY801770 |
| **189** | MVs/Palermo.ITA/21.16/2 [D8] | 3 | F | Hospital | OS+U | D8 | GROUP B | No | KY801734 |
| **190** | MVs/Palermo.ITA/22.16 [D8] | 7 | M | Hospital | OS+U | D8 | GROUP B | No | KY801735 |
| **191** | MVs/Trapani.ITA/23.16 [D8] | 1M | M | Community | U | D8 | GROUP B | No | KY801771 |
| **192** | MVs/Palermo.ITA/23.16 [D8] | 9M | F | Hospital | OS+U | D8 | GROUP B | No | KY801736 |
| **193** | MVs/Agrigento.ITA/24.16/1 [D8] | 5M | F | Community | OS+U | D8 | GROUP B | No | KY801711 |
| **194** | MVs/Palermo.ITA/24.16 [D8] | 2 | F | Hospital | OS+U | D8 | GROUP B | No | KY801737 |
| **195** | MVs/Agrigento.ITA/24.16/2 [D8] | 5 | M | Community | OS+U | D8 | GROUP B | No | KY801712 |
| **196** | MVs/Palermo.ITA/26.16/3 [D8] | 1 | F | Hospital | OS+U | D8 | GROUP B | No | KY801740 |
| **197** | MVs/Agrigento.ITA/26.16 [D8] | 1 | M | Hospital | OS+U | D8 | GROUP B | No | KY801713 |
| **198** | MVs/Palermo.ITA/26.16/1 [D8] | 8 | M | Hospital | OS+U | D8 | GROUP B | No | KY801738 |

| | | | | | | | | | |
|---|---|---|---|---|---|---|---|---|---|
| **199** | MVs/Palermo.ITA/26.16/2 [D8] | 3 | M | Hospital | OS+U | D8 | GROUP B | No | KY801739 |
| **200** | MVs/Palermo.ITA/29.16/1 [D8] | 21 | F | Community | OS+U | D8 | GROUP B | No | KY801742 |
| **201** | MVs/Palermo.ITA/29.16/2 [D8] | 26 | F | Community | OS+U | D8 | GROUP B | No | KY801743 |
| **202** | MVs/Palermo.ITA/28.16 [D8] | 1 | F | Hospital | OS+U | D8 | GROUP B | No | KY801741 |
| **203** | MVs/Trapani.ITA/29.16 [D8] | 25 | F | Community | U | D8 | GROUP B | No | KY801772 |
| **204** | MVs/Trapani.ITA/34.16 [D8] | 22 | M | Community | U | D8 | GROUP B | No | KY801773 |
| **205** | MVs/Trapani.ITA/39.16 [D8] | 41 | F | Community | U | D8 | GROUP B | No | KY801775 |
| **206** | MVs/Trapani.ITA/40.16/1 [D8] | 30 | M | Community | OS+U | D8 | GROUP B | No | KY801776 |
| **207** | MVs/Trapani.ITA/40.16/2 [D8] | 30 | F | Community | OS+U | D8 | GROUP B | No | KY801777 |
| **208** | MVs/Trapani.ITA/43.16 [D8] | 34 | F | Community | OS+U | D8 | GROUP B | No | KY801778 |
| **209** | MVs/Catania.ITA/6.17 [D8] | 25 | F | Hospital | OS+U | D8 | GROUP A | Yes | KY801718 |
| **210** | MVs/Enna.ITA/13.17/1 [D8] | 26 | M | Hospital | U | D8 | GROUP A | No | Submitted |
| **211** | MVs/Agrigento.ITA/17.17 [D8] | 35 | F | Hospital | U | D8 | GROUP A | No | Submitted |
| **212** | MVs/Agrigento.ITA/18.17/1 [D8] | 25 | F | Hospital | U | D8 | GROUP A | No | Submitted |
| **213** | MVs/Agrigento.ITA/18.17/2 [D8] | 44 | F | Community | U | D8 | GROUP A | No | Submitted |
| **214** | MVs/Agrigento.ITA/19.17/1 [D8] | 11M | M | Community | U | D8 | GROUP A | No | Submitted |
| **215** | MVs/Agrigento.ITA/20.17 [D8] | 24 | F | Hospital | U | D8 | GROUP A | No | Submitted |
| **216** | MVs/Caltanissetta.ITA/20.17 [D8] | 22 | M | Community | OS+U | D8 | GROUP A | No | Submitted |
| **217** | MVs/Catania.ITA/27.17/7 [D8] | 9 | M | Hospital | OS+U | D8 | | No | Submitted |
| **218** | MVs/Siracusa.ITA/29.17 [D8] | 32 | M | Hospital | OS+U | D8 | GROUP A | No | Submitted |
| **219** | MVs/Siracusa.ITA/31.17 [D8] | 32 | F | Hospital | OS+U | D8 | GROUP A | No | Submitted |
| **220** | MVs/Catania.ITA/12.16 [H1] | 44 | F | Community | OS+U | H1 | | No | KY801715 |
| **221** | MVs/Catania.ITA/11.16 [H1] | 54 | M | Hospital | OS+U | H1 | | No | KY801714 |

**Legend:**

OS Oro-pharyngeal swab

U Urine
